# Delayed Recognition of Wernicke's Encephalopathy Following the Interruption of Thiamine in an Alcohol-Dependent Patient With Status Epilepticus: A Case Report

**DOI:** 10.7759/cureus.106470

**Published:** 2026-04-05

**Authors:** Dia Mobaideen, Gihad Osman, Jehad Shitawi

**Affiliations:** 1 Acute Medicine, Lincoln County Hospital, Lincoln, GBR; 2 Geriatrics, Lincoln County Hospital, Lincoln, GBR; 3 Emergency Medicine, Lincoln County Hospital, Lincoln, GBR

**Keywords:** alcohol misuse, mri of the brain and spine, refractory seizure, status epilepticus (se), wernicke's encephalopathy (we)

## Abstract

Wernicke's encephalopathy (WE) is an acute neurological disorder precipitated by thiamine deficiency, most commonly associated with chronic alcohol misuse. WE is underdiagnosed and can lead to significant neurological morbidity if treatment is delayed. We report the case of a 35-year-old man with alcohol dependence and a history of seizures who was admitted for the management of status epilepticus requiring intubation and intensive care. During his sedation, his regular thiamine supplementation was inadvertently withheld for four days. Following extubation and seizure stabilization, the patient developed confusion and nystagmus, raising suspicion of WE. Thiamine was promptly reinitiated empirically. Subsequent magnetic resonance imaging (MRI) revealed findings consistent with WE. The patient exhibited gradual neurological improvement with high-dose thiamine therapy and rehabilitation and was eventually discharged. This case underscores the importance of maintaining thiamine supplementation in high-risk patients and emphasizes the need to initiate treatment based on clinical suspicion rather than waiting for confirmatory neuroimaging.

## Introduction

Wernicke's encephalopathy (WE) is a potentially reversible neurological emergency precipitated by thiamine (vitamin B1) deficiency and is a frequently overlooked diagnosis. Most commonly associated with chronic alcohol misuse, WE can also develop due to malnutrition, prolonged vomiting, critical illness, and other conditions causing nutritional depletion [1].

Thiamine serves as an essential cofactor in carbohydrate metabolism and neuronal energy production. The depletion of thiamine stores leads to neuronal injury in metabolically vulnerable areas of the brain, including the mammillary bodies, thalami, and periaqueductal regions.

WE is characterized by a classical triad of confusion, ocular abnormalities (nystagmus or ophthalmoplegia), and gait ataxia. However, the complete triad is present in only a minority of patients, making early diagnosis challenging [2]. Because diagnostic delay can lead to irreversible neurological damage or progression to Korsakoff syndrome, early empirical treatment with parenteral thiamine is recommended upon clinical suspicion of WE.

## Case presentation

A 35-year-old man with a history of chronic alcohol dependence and epilepsy presented to the emergency department following an episode of generalized tonic-clonic status epilepticus. Collateral history indicated heavy alcohol consumption in the days preceding his admission. The patient was known to be prescribed regular oral thiamine for the management of his alcohol dependence.

Despite the administration of benzodiazepines on arrival, the patient continued to experience recurrent seizures. Due to persistent seizure activity and the need for airway protection, he was intubated and transferred to the intensive care unit (ICU) for further management.

While in the ICU, the patient was sedated, and status epilepticus was controlled using antiepileptic therapy. During the initial period of sedation and mechanical ventilation, several of his regular oral medications were withheld. Unfortunately, his routine thiamine supplementation was also discontinued during this period and was not replaced with a parenteral equivalent.

Seizure activity was successfully controlled over the following days, and the patient's neurological status stabilized. Sedation was gradually weaned, and the patient was extubated after four days of ICU care.

Following extubation, the patient exhibited persistent confusion and disorientation, with impaired attention and fluctuating levels of alertness. Neurological examination revealed horizontal nystagmus. Post-ictal confusion was initially considered; however, the persistence of symptoms combined with the presence of ocular signs raised clinical suspicion for WE, particularly given the patient's history of chronic alcohol misuse and the recent interruption of thiamine therapy.

Given the high clinical suspicion, intravenous thiamine therapy was immediately initiated without awaiting radiological confirmation. 

Magnetic resonance imaging (MRI) findings

**Figure 1:**
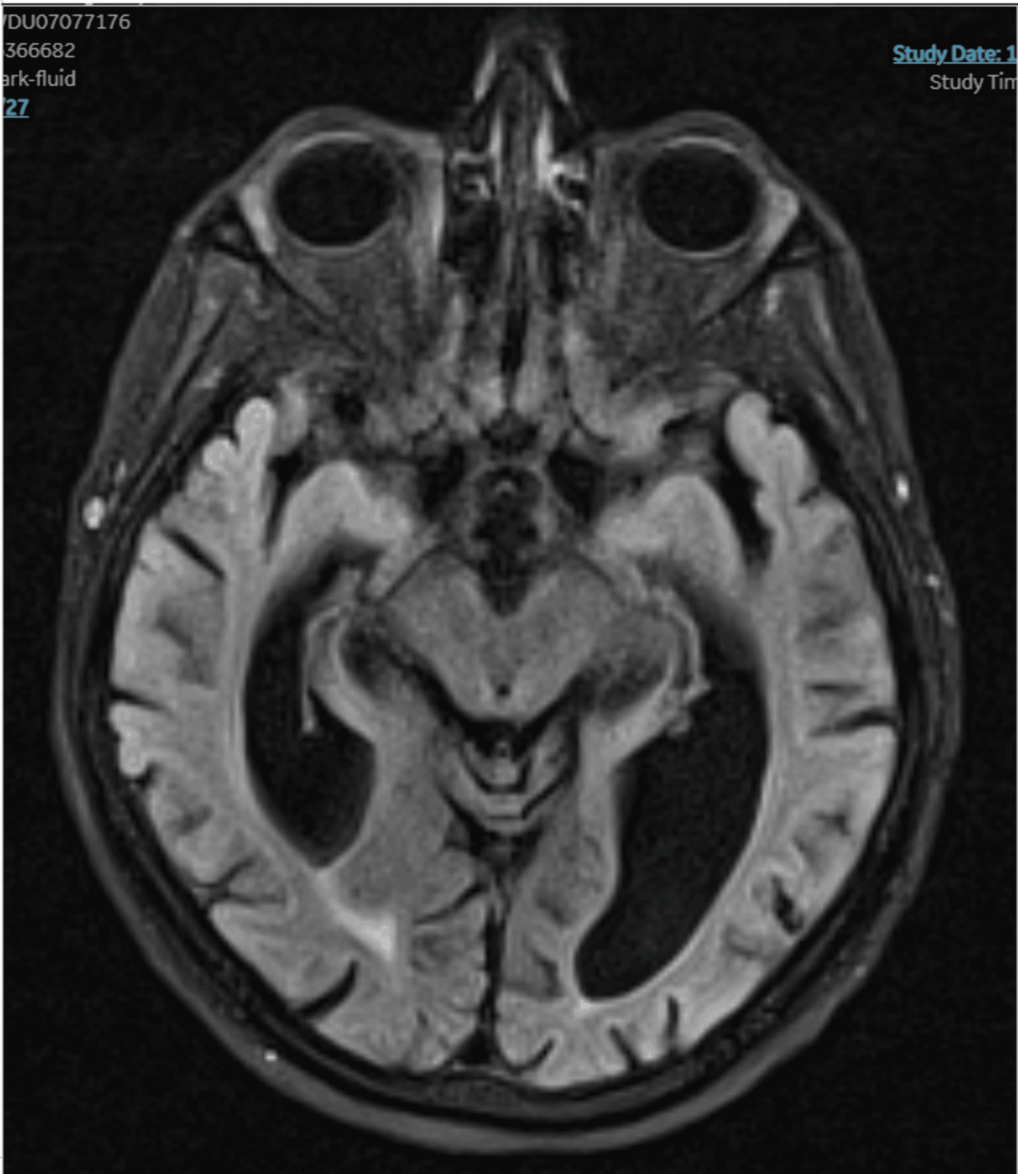
T2 FLAIR hyperintensities in the mammillary bodies and periaqueductal gray matter Axial T2-weighted FLAIR magnetic resonance imaging of the brain demonstrates symmetrical hyperintense signal abnormalities involving the medial thalami, mammillary bodies, and periaqueductal gray matter, which are characteristic regions affected in Wernicke's encephalopathy. These findings support the clinical diagnosis in the context of thiamine deficiency risk factors and compatible neurological symptoms. The imaging was obtained during the patient's acute hospitalization. No contrast enhancement was required for diagnostic interpretation. FLAIR: fluid-attenuated inversion recovery

MRI of the patient's brain was performed (Figure 1). The imaging revealed symmetrical signal abnormalities in regions classically associated with WE, including areas of signal hyperintensity on T2-weighted and fluid-attenuated inversion recovery (FLAIR) sequences involving the medial thalami, mammillary bodies, and periaqueductal grey matter. These radiological findings were considered highly suggestive of WE, supporting the clinical diagnosis. 

Clinical course and outcome

Following the initiation of high-dose intravenous thiamine therapy, the patient exhibited gradual neurological improvement. His confusion progressively diminished over several days, and the nystagmus became less prominent. Once stable, the patient continued receiving parenteral thiamine, followed by oral supplementation. He was transferred from the acute medical unit to a rehabilitation setting to support recovery and functional improvement.

During the rehabilitation period, the patient's cognitive function continued to improve, and seizure control was maintained with antiepileptic medication. He was eventually discharged with ongoing oral thiamine supplementation, antiepileptic therapy, a scheduled follow-up with neurology services, and a referral to alcohol support and rehabilitation services.

## Discussion

WE remains a significantly underdiagnosed neurological emergency despite its well-established association with alcohol misuse. Autopsy-based studies by Harper et al. demonstrated that a large proportion of cases were not recognized clinically, with many identified only post-mortem [2]. Subsequent epidemiological analyses suggest that up to 80% of cases may be missed during life, highlighting a substantial diagnostic gap [2]. This under-recognition is clinically important, as untreated WE may progress to Korsakoff syndrome, resulting in irreversible cognitive and memory deficits.

The pathophysiology of WE is driven by thiamine deficiency, which impairs cerebral energy metabolism. Thiamine is an essential cofactor for key enzymes such as pyruvate dehydrogenase, α-ketoglutarate dehydrogenase, and transketolase, which are critical in carbohydrate metabolism and adenosine triphosphate (ATP) production. Deficiency leads to mitochondrial dysfunction, lactate accumulation, oxidative stress, and neuronal injury, particularly in metabolically active regions such as the mammillary bodies, thalami, periaqueductal grey matter, and cerebellum [3]. Experimental studies also suggest that glutamate-mediated excitotoxicity contributes to selective neuronal vulnerability. Patients with alcohol dependence are particularly susceptible due to poor nutritional intake, impaired gastrointestinal absorption, reduced hepatic storage, and altered thiamine utilization. However, WE is increasingly recognized in non-alcoholic populations, including critically ill, malnourished, or post-bariatric surgery patients [3].

Clinical diagnosis remains challenging due to variable presentation. Victor et al. reported that the classical triad of confusion, ataxia, and ophthalmoplegia occurs in only 10-16% of cases [3]. Many patients present with incomplete or atypical features, contributing to frequent misdiagnosis or delayed treatment. To improve sensitivity, Caine et al. proposed operational diagnostic criteria incorporating dietary deficiency, ocular abnormalities, cerebellar dysfunction, and altered mental state or memory impairment, which have been shown to detect more cases than reliance on the classical triad alone [4].

Neuroimaging can provide supportive evidence but lacks sufficient sensitivity to exclude the diagnosis. Antunez et al. described the typical MRI findings of symmetrical signal abnormalities in the thalami, mammillary bodies, periaqueductal grey matter, and tectal plate [5]. Zuccoli et al. later reported additional atypical patterns, including cortical and cerebellar involvement, especially in non-alcoholic patients [6]. However, studies estimate that 40-50% of patients may have normal imaging, reinforcing the principle that treatment should be initiated based on clinical suspicion rather than radiological confirmation.

This case also highlights a critical patient safety issue: inadvertent interruption of essential nutritional supplementation during acute care. Guidelines from the Royal College of Physicians and Thomson et al. emphasize the importance of early parenteral thiamine replacement in all at-risk patients, particularly when oral intake is suspended [7]. Failure to provide adequate supplementation can rapidly precipitate neurological injury, yet gaps in practice remain common, underscoring a preventable cause of morbidity and highlighting opportunities for improving inpatient care. 

Limitations

This report has several limitations inherent to a single case study. Some clinical details, including precise pre-admission thiamine dosing, adherence to supplementation, and quantification of alcohol intake, were not reliably documented in the available medical records and therefore could not be reported. Similarly, laboratory markers that may support thiamine deficiency, such as serum thiamine levels or erythrocyte transketolase activity, were not obtained during the acute presentation. In addition, although MRI findings supported the clinical diagnosis, not all radiological sequences were available for detailed analysis. Finally, as with all case reports, causal relationships cannot be definitively established, and the observations described should be interpreted within the context of a single patient. Despite these limitations, the case highlights an important and preventable clinical scenario in which interruption of thiamine supplementation during critical care may contribute to the development of WE in high-risk patients.

## Conclusions

This case highlights the importance of maintaining a high index of suspicion for WE in patients with alcohol dependence who develop new neurological symptoms during hospital admission. In this patient, interruption of thiamine supplementation during ICU management may have contributed to the development or unmasking of WE. The case underscores the importance of maintaining essential nutritional supplementation in high-risk patients and initiating empirical thiamine therapy when clinical suspicion arises, rather than delaying treatment while awaiting imaging. It also highlights medication reconciliation during ICU admissions as an important patient safety consideration.
